# Supplementary figures and images for: The Contributions of Sub-Communities to the Assembly Process and Ecological Mechanisms of Bacterial Communities along the Cotton Soil–Root Continuum Niche Gradient
Source: Microorganisms. 2024 Apr 26;12(5):869. doi: 10.3390/microorganisms12050869 (PMC11123189; doi:10.3390/microorganisms12050869)

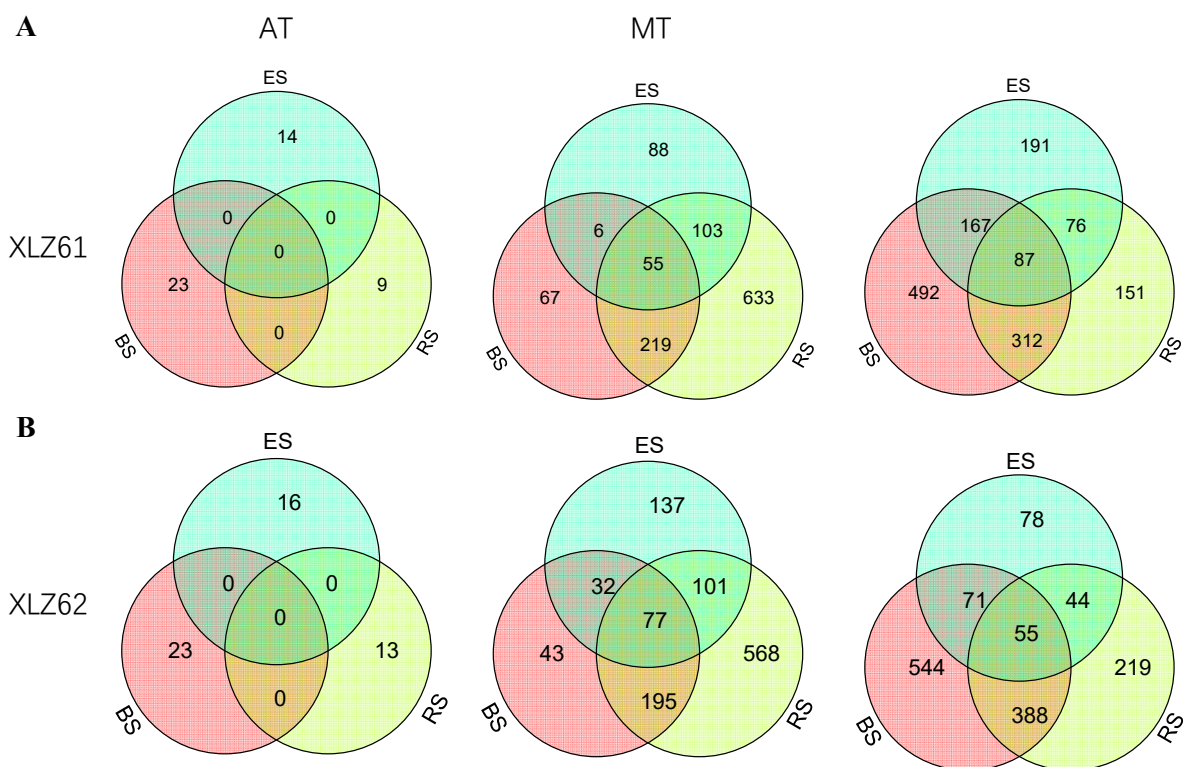

Fig S1

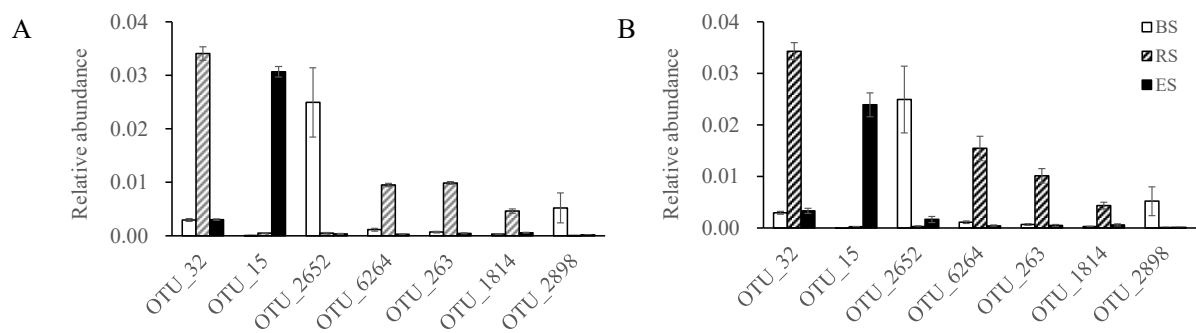

Fig S2

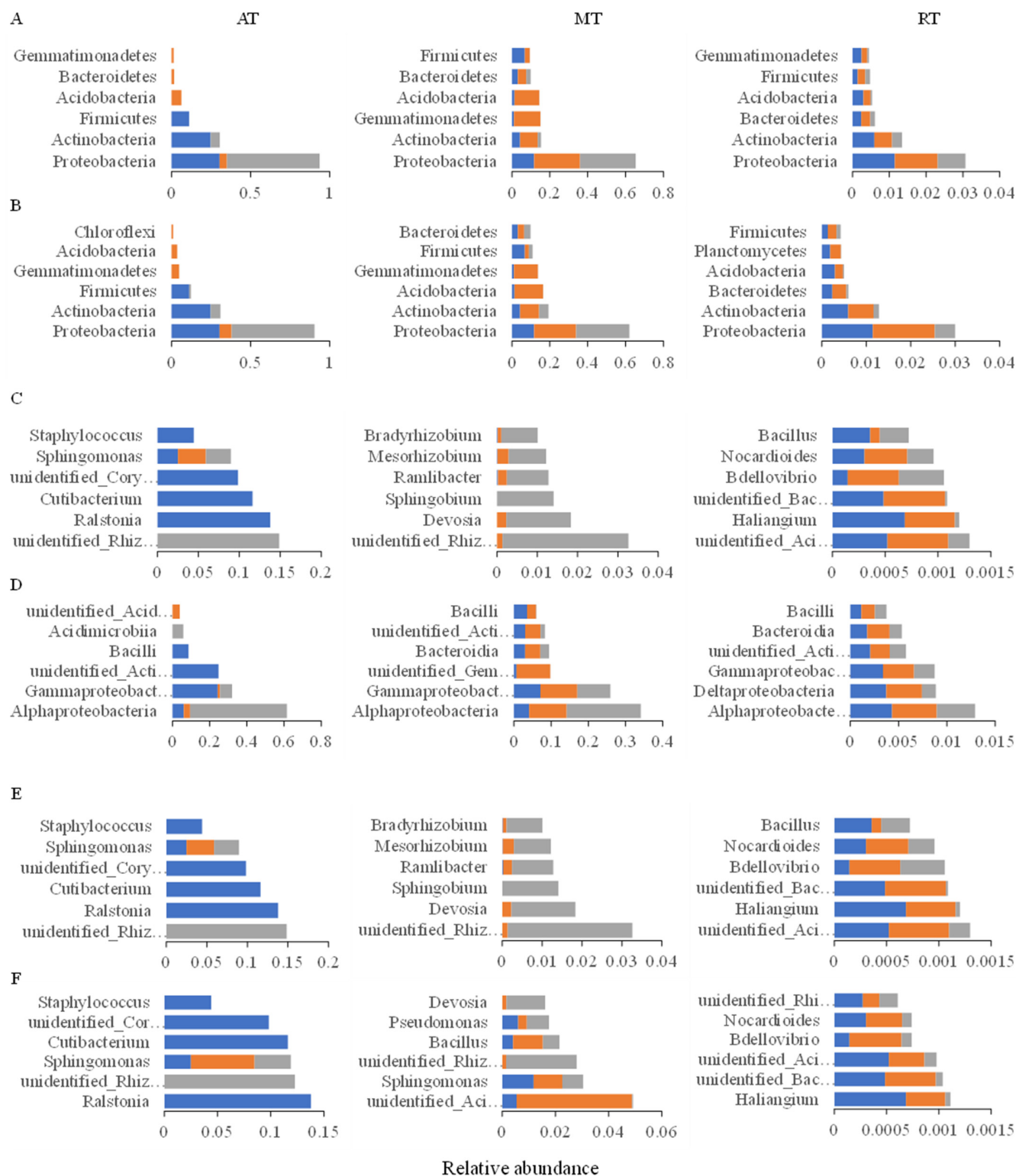

Fig S3

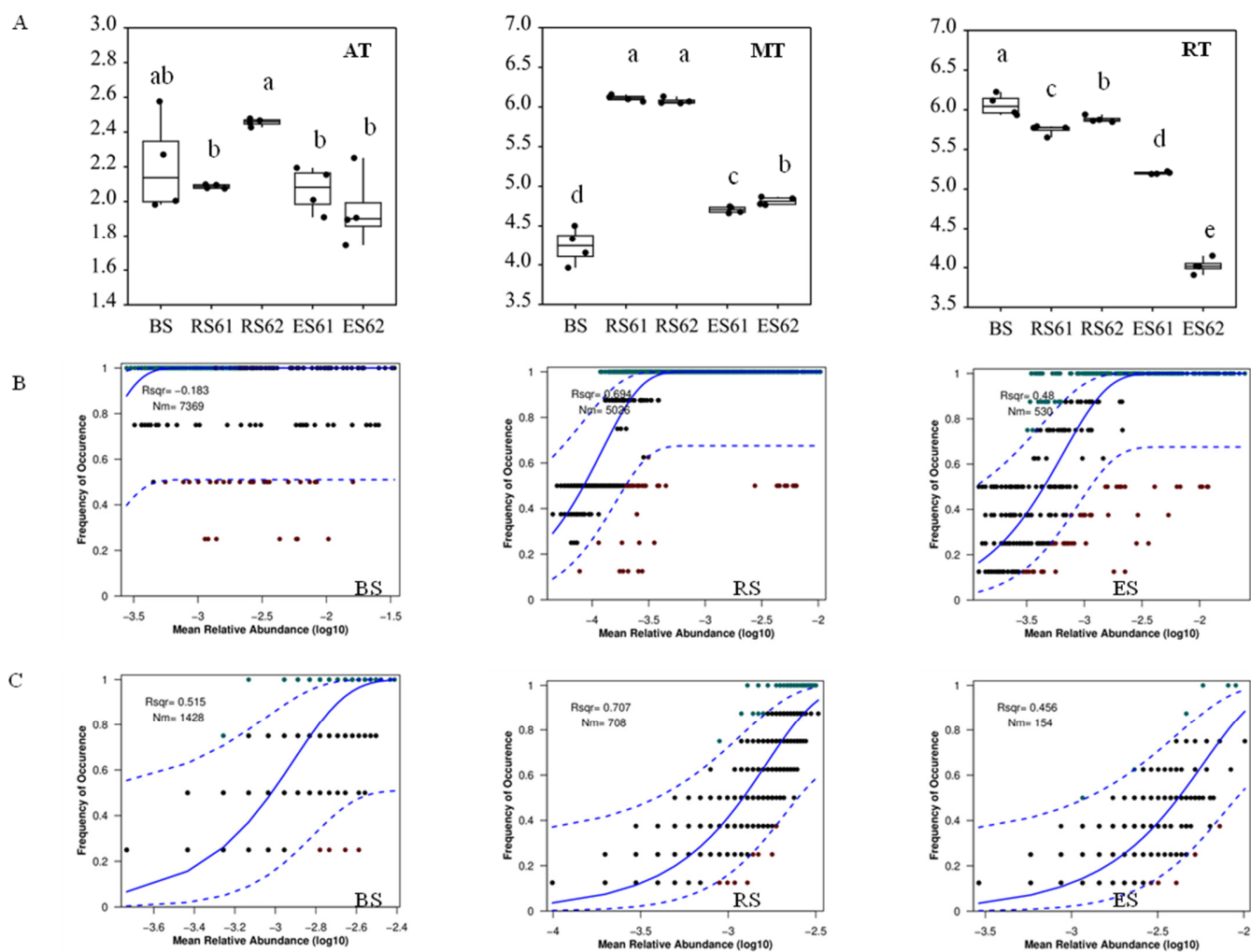

Fig S4

Supplement: Supplementary file 1 [file microorganisms-12-00869-s001.zip › microorganisms-2934010-supplementary.pdf]
